# Public data sources for regulatory genomic features

**DOI:** 10.1515/medgen-2021-2075

**Published:** 2021-08-14

**Authors:** Samuele Garda, Jana Marie Schwarz, Markus Schuelke, Ulf Leser, Dominik Seelow

**Affiliations:** Knowledge Management in Bioinformatics, Institute for Computer Science, Humboldt-Universität zu Berlin, Unter den Linden 6, 10099 Berlin, Germany; Department of Neuropediatrics, Charité–Universitätsmedizin Berlin, Corporate Member of Freie Universität Berlin and Humboldt-Universität zu Berlin, Berlin, Germany; NeuroCure Cluster of Excellence, Charité–Universitätsmedizin Berlin, Corporate Member of Freie Universität Berlin and Humboldt-Universität zu Berlin, Berlin, Germany; BIH–Bioinformatics and Translational Genetics, Berlin Institute of Health at Charité-Universitätsmedizin Berlin, Berlin, Germany; Institute for Medical and Human Genetics, Charité–Universitätsmedizin Berlin, Corporate Member of Freie Universität Berlin and Humboldt-Universität zu Berlin, Berlin, Germany

**Keywords:** non-coding genome, functional DNA elements, databases, text mining, information extraction

## Abstract

High-throughput technologies have led to a continuously growing amount of information about regulatory features in the genome. A wealth of data generated by large international research consortia is available from online databases. Disease-driven studies provide details on specific DNA elements or epigenetic modifications regulating gene expression in specific cellular and developmental contexts, but these results are usually only published in scientific articles. All this information can be helpful in interpreting variants in the regulatory genome. This review describes a selection of high-profile data sources providing information on the non-coding genome, as well as pitfalls and techniques to search and capture information from the literature.

## Introduction

Over the course of the previous three decades, research on the molecular basis of monogenic disorders has focused on the coding part of the human genome. This was based on the assumption that changes in an individual’s phenotype are primarily due to functional changes in proteins, which are predominantly caused by variants in the protein-coding DNA sequence. Single-gene sequencing was easy using the Sanger method.

However, despite years of research, many genetic disorders still cannot be traced back to changes in the coding sequence. This also applies to many “common” diseases, such as diabetes and hypertension. Accordingly, there has been an increased interest in the non-coding part of the genome, especially in DNA elements regulating gene expression, such as transcription factor binding sites (TFBSs), promoters, or enhancers (Krude et al. this edition, Leiz et al. this edition, Guo et al. this edition). The underlying hypothesis is that changes in the temporal and spatial order of gene expression can have a physiological impact that is as severe as changes in protein structure and function and lead to disease.

This change in focus was accompanied by rapid advances in the experimental techniques available to geneticists (Leiz et al. this edition, Guo et al. this edition). The invention of next-generation sequencing (NGS) [1] about 13 years ago enabled affordable whole exome sequencing (WES), covering approximately 2 % of the genome. Whole genome sequencing (WGS), however, remained relatively expensive. This situation is now changing, due to both falling prices of WGS and the availability of new and targeted assays addressing specific mechanisms that play a role in gene regulation, such as the assay for transposase-accessible chromatin using sequencing (ATAC-seq), chromatin immunoprecipitation coupled with sequencing (ChIP-seq), and massively parallel reporter assays (MPRA). These developments led to the accumulation of a wealth of data on different regulatory features in the non-coding part of the human genome, often driven by large international research initiatives (Leiz et al. this edition). These data are available to practitioners to derive or test hypotheses on the molecular basis of diseases in cases where WES had not led to a molecular diagnosis.

The paradigms underlying data acquisition can be divided into **(i)** large-scale hypothesis-free and genome-wide approaches, e. g., genome-wide ChIP-seq analyses, and **(ii)** hypothesis- or disease-driven studies. While the former approaches measure the state of thousands of regulatory features in a given model system in a single experiment, their read-outs have often been determined under standard cell culture conditions and not during specific cellular functional states, e. g., during the developmental phase of a tissue *versus* its adult stage (see Krude et al. this edition). In contrast, the latter type of experiments focus on the effects of a subset of features on a small set of candidate genes in a specific setting, often reaching higher pathophysiological relevance at the price of a much lower throughput.

Researchers can access data from the first group of acquisition methods *via* large web-based databases. In addition, metadatabases provide integrated access to data of different types and from different studies. Results from the second group of studies are predominantly published in textual form in the scientific literature. Accessing this information requires either extensive reading or advanced information management tools, such as search engines and information extraction (IE) algorithms. A third means of data access is the use of curated (meta)databases, which are built by manually processing publications and turning their content into a structured form. The *OMIM*^1^ database for Mendelian diseases and disease genes [2] is a prominent example for such a curated database.

In this review, we give an overview of prominent data sources for studying non-coding regulatory features in the human genome with the goal to establish and test hypotheses on their role in genetic disorders. We focus on tools and systems that are freely available for non-commercial use. While many of those resources are intuitively usable by non-IT experts, some, especially those for IE, require a thorough understanding of computational concepts and programming skills. These resources provide the base for future methods of information search and analysis also for non-IT users.

## Regulatory genomic features

Regulatory genomic features can be divided into different subclasses: **(i)** those that are encoded in the DNA sequence *per se*, e. g., TFBSs, and **(ii)** those that are modifications of the DNA or its protein “backbone,” e. g., the histones of the chromatin. Table 1 lists some prominent regulatory features.

Few of these features exert their function in isolation. Eukaryotic gene expression requires not only signals in the DNA sequence, such as transcriptional start sites or splicing signals, but a whole set of DNA:DNA, DNA:protein, and protein:protein interactions in the vicinity of a gene. For instance, the binding of a transcription factor (TF) to a promoter is necessary, but not sufficient, for gene expression. The binding of additional factors, often interacting with each other, induces further changes in the chromatin structure needed to make the DNA “accessible” for the transcribing enzymes (Leiz et al. this issue). This process is additionally controlled by enhancers, DNA elements that may be located hundreds of kilobases away from the promoters with which they interact (Krude et al. this issue, Leiz et al. this issue). Although such mechanisms have been known for decades [3], only the recent technical advances have made their systematic and genome-wide study feasible (Guo et al. this issue).

The functional annotation of regulatory features and their description is less straightforward than for the coding DNA sequence. Humans have only two alleles of each genomic region and the genome stays stable over time, except for somatic mutations. The DNA sequence of an individual is comparably easy to record, and standard formats for data exchange have been established, e. g., variant call format (*.vcf) files. This is not the case for the “regulome”. Although regulatory features encoded by the DNA itself, e. g., TFBSs, remain stable across cells, time, and physiological states, their accessibility and function, however, do not. In addition, there are transient modifications such as chromatin modifications or DNA methylation. Therefore, regulatory processes cannot be described in a dichotomous fashion, e. g., as being present or absent, but require quantitative read-outs, such as the abundance of a TF in a cell or its binding affinity and the ensuing effect on the phenotype, such as the protein abundance (see Leiz et al. this issue).

**Table 1:** **Regulatory features**. The table shows a selection of regulatory features in the genome along with some of the associated biological roles and experimental techniques to detect them. Please note that the functions of many regulatory features are not yet fully determined and still a matter of scientific debate.

Element class	Involved molecule class	Element	Location/biological role	Assays/methods
TFBS	DNA	TFBS	specific sequences in the DNA typically found at promoters, enhancers, silencers; gene activation or repression	ChIP-seq/-exo/-nexus, MPRA
Histone modifications	DNA-bound protein	H3K4me1, H3K4me3, H3K27ac, H3K9me3, H3K27me, and others	around enhancers, promoters, silencers; gene activation or repression	ChIP-seq/-exo/-nexus
CTCF binding site	DNA	CTCF binding site	often located at TAD boundaries where the insulator function of CTCF together with cohesion regulates genome topology	ChIP-seq/-exo/-nexus
Open chromatin regions	DNA and DNA-bound protein	DNaseI-hypersensitive site	found at transcriptionally active sites; gene activation	DNaseI-seq, MNase-seq, ATAC-seq
DNA methylation	DNA	5mC	methylation of clusters of CpG dinucleotides found in regulatory elements (CpG islands) affects gene expression	reduced representation (RRBS) or whole genome bisulfite sequencing (WGBS)
Genome-wide DNA:DNA interactions	DNA	interacting sites	occurs between enhancers, silencers, and promoters; gene activation or repression	5C, Hi-C, GAM

**Abbreviations**: 5mC, 5-methylcytosine; ChIP, chromatin immunoprecipitation; CTCF, CCCTC-binding transcription factor; GAM, genome architecture mapping; MPRA, massive parallel reporter assay; TAD, topologically associating domain; TFBS, transcription factor binding site.

## Accessing regulatory information from databases

Despite the aforementioned difficulties, researchers and research consortia have started to annotate the non-coding part of the genome. High-profile projects started around 10 years ago and include *ENCODE*^2^ [4], *BluePrint*^3^ [5], *FANTOM*^4^ [6], *GTEx*^5^ [7], and *Roadmap Epigenomics*^6^ [8]. Table 2A lists a selection of such projects, some regulatory features they focus on, and the experimental techniques used for data acquisition. Each consortium provides detailed information about their methods and the data on their respective websites.

These large, publicly funded projects make their data freely available in web-based databases. These can be used in two different ways. **(i)** Selected subsets of the data can be downloaded in their raw form. Such raw data are only recommended for specialists, because the necessary processing steps, such as data normalization and quality control, are non-trivial and require bioinformatic skills. **(ii)** Users may access pre-processed and quality-filtered datasets using the interactive web interfaces of the databases. Interfaces are typically constructed to make intuitive access possible and include tools for data visualization, links to other databases, and often some limited analysis methods. These web interfaces reach their limits when information from different data sources needs to be combined, e. g., for the joint analysis of TF binding activities and histone modifications in a given cell type. Such an integration requires careful statistical data processing.

**Figure 1:**
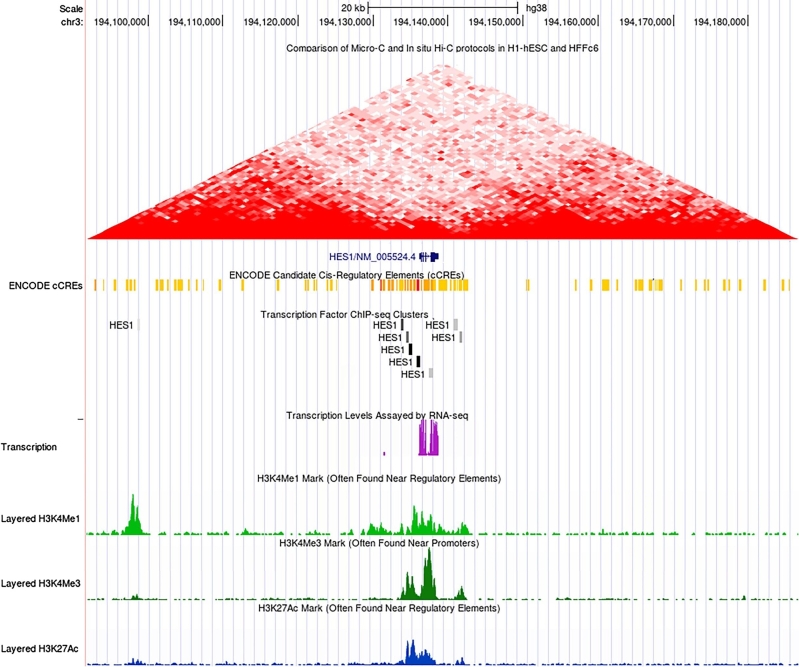
**The UCSC browser**. The screenshot of the browser depicts the regulatory landscape of the human *HES1* gene with data on Hi-C interaction, *cis*-regulatory elements (CREs; red: promotors; orange: enhancers), TFBSs (here for Hairy and Enhancer of Split 1 transcription factor [HES1]), mRNA transcript levels, and histone marks (H3K4me2 and H3K4me2: active regulatory regions; H3K27Ac: active distal and proximal regulatory regions). The browser can be customized to depict the regulatory features from certain cell lines, here from human skeletal muscle myoblasts (HSMMs), which have been investigated in the *ENCODE* project. The *HES1* gene inhibits its own transcription by the binding of its gene product on HES1-TFBSs in the vicinity of the gene (see Leitz et al. this issue).

An alternative and more accessible way is offered by (meta)databases, such as *Ensembl*^7^ [9] or *UCSC*^8^ [10] (Table 2B). These databases assemble data from different studies, integrate them into a common schema, and perform comprehensive normalization to achieve comparability of results. Both projects provide browser-based interfaces for data access called *Ensembl Genome Browser* and *UCSC Genome Browser*. In the UCSC browser, users can inspect the promoter region of a particular gene by entering either the gene’s coordinates or its gene symbol. The browser will display a collection of “data tracks” with different features in a window centered around the gene. Non-coding regulatory DNA elements such as enhancers from the *ENCODE* project can be depicted alongside the coding sequence of the gene of interest. Figure 1, as an example, depicts histone modifications, TFBSs, and other regulatory features from different experiments in different cell lines. In addition to a classical browser, *Ensembl* also offers *Biomart*,^9^ an interface for efficient access by computer programs [11].

**Table 2:** **Large scale studies and databases. (A)** A selection of large-scale research collaborations with the aim to elucidate form and function of the non-coding part of the genome as well as the epigenome. **(B)** A selection of metadatabases providing information from different data sources. Graphical interfaces enable users to conveniently browse through the data. **(C)** A selection of databases with information derived from automated or curated literature search.

**A: Large scale studies**			
**Name**	**Element class**	**Technique**	**Year [start]**
ENCODE^10^	TFBS, histone modifications, genome-wide DNA:DNA interactions, and others (Figure 2A)	ChIP-seq, 5C, Hi-C, DNaseI-seq, and many more	2012
Roadmap Epigenomics^11^	TFBS, histone modifications, DNA methylation, transcribed regions, and others (Figure 2A)	ChIP-seq, DNaseI-seq, WGBS, RNA-seq, and many more	2015
FANTOM^12^	FANTOM5 & 6: promoters, enhancers, lncRNAs, and miRNAs	CAGE, deepCAGE, other CAGE methods, full-length cDNA technology	2014
**B: Metadatabases**			
**Name**	**Element classes included**	**Databases included**	
Ensembl Regulation^13^	TFBS, CTCF binding sites, TSS, miRNA target sites; annotation of open chromatin, promoters, enhancers, and others	ENCODE, FANTOM5, DianaTarBase, VISTA, and more	
UCSC^14^	TFBS, histone modifications, DHS, CpG islands, DNA:DNA interactions, sno/miRNA target sites, promoters, enhancers, and others	ENCODE, ORegAnno, GeneHancer, VISTA, and more	
**C: Literature-derived databases**			
**Name**	**Curation**	**Element classes included**	
EnDB^15^	human	Enhancer	
EnDisease^16^	human	Enhancer	
DiseaseEnhancer^17^	human	Enhancer	
JASPAR^18^	human	TFBS	
GTDR^19^	human	TFBS	
ORegAnno^20^	human, semi-automatic	TFBS	
RegulomeDB^21^	human	TFBS, promoters	

**Abbreviations**: CTCF: CCCTC-binding transcription factor; ChIP: chromatin immunoprecipitation; DHS: DNaseI-hypersensitive site; lncRNA: long non-coding RNA; miRNA: micro RNA; snoRNA: small nucleolar RNA; TFBS: transcription factor binding site; TSS: transcription start site; WGBS: whole genome bisulfite sequencing.

**Figure 2:**
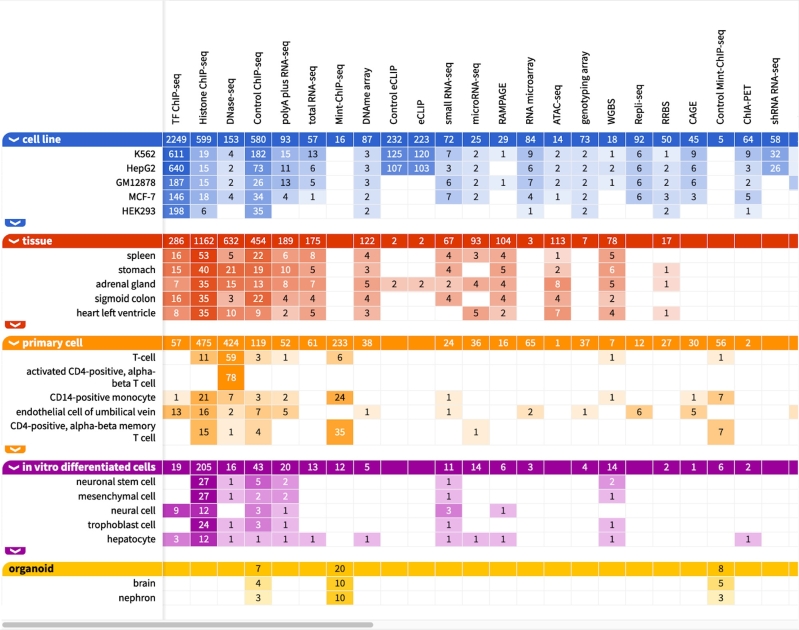
**The ENCODE browser**. This screenshot depicts a selection of some of the different assays that were used to create the data accessible *via* the *ENCODE* browser. Please note that there are many more columns than those shown here.

Other integrated public data sources focus on specific qualitative aspects of specific types of regulatory features. An important source for information on TFs is *JASPAR* [12], which is a freely accessible collection of data on TF binding collected from various databases and the literature. *JASPAR* is curated, meaning that all data items are evaluated by experts before inclusion into the database. Though this process is costly and delays the addition of new data, it ensures higher data quality. A list of important curated databases can be found in Table 2C.

Though the *in-silico* prediction of the effects of DNA variants within regulatory elements is still in its infancy, displaying the “context” of such variants is feasible and informative. Tools such as the *Ensembl Variant Effect Predictor* [13], *RegulationSpotter* [14], or *RegulomeDB* [15] analyze if a given DNA variant resides within a regulatory region and offer information and predictions about its potential effect (see Schwarz et al. this edition).

## Accessing literature-based data

In contrast to the large-scale public databases focusing on high-throughput experiments, the results of targeted, small-scale experiments are published mainly in scientific articles. Finding relevant information for a given patient requires two steps: **(i)** identifying the most relevant articles and **(ii)** finding the most relevant information within those articles. Currently, this process cannot be fully automated: Whereas the search for relevant articles is supported by medical article databases and domain-specific search engines, finding the most relevant information within such an article must be accomplished by human reading. We describe two popular search engines for finding articles relevant for a given case as well as ongoing research towards the automatic extraction of specific types of medical knowledge from articles. The latter has the potential to significantly reduce the time spent on literature review, but requires advanced computational skills. Applying automatic methods to large collections of articles, usually followed by expert curation, led to the creation of literature-derived knowledge databases. These are the third type of data source for information on regulatory elements in the non-coding genome described below.

### Search engines

The search within large collections of digitized documents is supported by popular search engines, such as Google© or Bing©. These general-domain search engines are poorly suited for searching within collections of scientific articles, which have a fixed structure, undergo quality control through peer review, and often use highly specialized vocabulary. Searching such collections is the realm of domain-centered search engines such as *PubMed*^22^ [16], which indexes all abstracts from the *Medline* service and makes them searchable through a powerful query language. Another search engine for *Medline* abstracts is *Pubtator*^23^ [17]. *PubTator* performs a pre-processing of articles to recognize and normalize mentions of important biomedical entities.

#### PubMed

*PubMed* is a publicly accessible web service for user-friendly search within the *Medline* collection of scientific articles. It contains and searches the abstract, title, authors, and keywords for most articles. The abstract is often missing for articles published prior to the 1980s. The full text is linked, but not indexed for ∼21 million articles. Of these, ∼6.7 million full text articles are freely available, while the rest require subscriptions. *PubMed* indexed more than 31 million articles from more than 5,000 journals in fall 2020.

**Figure 3:**
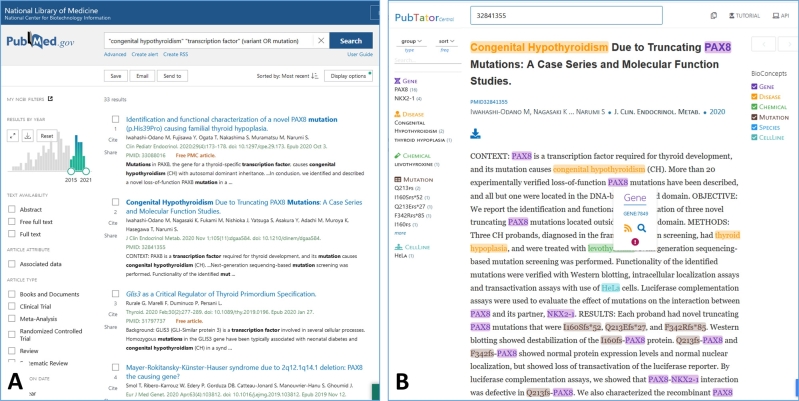
**PubMed and PubTator. (A)** Result of a *PubMed* search using multiple keywords combined by AND (default) or OR and a restriction on the year of publication (small figure at middle left). **(B)** Display of the second search result from **panel A** in *Pubtator*. Recognized entities are marked by different colors and are linked to other databases for further information.

*PubMed* can be searched either by keywords or using special Boolean syntax to define constraints on the set of matching articles, e. g., publication year, journal name, or authors. Articles are linked to their respective journal homepages and to other articles that have a similar content (see the example in Figure 3A). A typical search starts with general terms, often resulting in thousands of matches, that are iteratively narrowed down by adding further search terms or constraints. Because articles in *PubMed* are annotated with standardized keywords from the list of *Medical Subject Headings* (MeSH), this can be used within searches to better cope with synonyms, homonyms, or hyperonyms. Search results are either ranked by a score reflecting the quality of the match of an article to the query or by date of publication.

A recent unconstrained search for “congenital hypothyroidism” (see Krude et al. this issue) resulted in 5,577 articles. Refining this search to publications also mentioning “transcription factors” reduced this set to 153 articles. Further constraint to articles published within the last 5 years and containing either the term “mutation” or “variant” led to a set of 33 highly specific articles (0.6 % of the original 5,577 articles). During such a search process, *PubMed* performs a number of optimizations. For instance, it automatically detects certain spelling variations of search terms, e. g., “mutation” or “mutations,” and performs a limited type of logical reasoning by using information encoded in MeSH. For instance, MeSH annotates articles mentioning the term “GATA6” with the concept “transcription factor.” Accordingly, a search for the term “transcription factor” also returns articles containing the term “GATA6” even if the term “transcription factor” is not mentioned in particular (see Figure 3A).

#### Pubtator

*Pubtator* is the prototype of a next-generation search engine offering a glimpse into the improvement that can be accomplished by modern text-mining algorithms [17]. *Pubtator* indexes a pre-processed *Medline* version, in which specialized algorithms detect and annotate all mentions of a set of important biomedical entities, namely genes, chemicals, diseases, species, mutations, and cell lines. Mentions are mapped to entity type-specific databases offering further information, e. g., each detected gene is mapped to the *NCBI gene database*.^24^ By means of this mapping, *Pubtator* is capable of searching over synonyms, i. e., multiple names for the same entity, and of disambiguating homonyms, entities with the same name, but different context-depending meanings, in a significantly more comprehensive manner than possible with MeSH. Figure 3B shows the *Pubtator* view of the article that is second in the result list on Figure 3A. The term “PAX8” is recognized as a gene name. Hovering over the colored mention, a pop-up shows the corresponding NCBI Gene ID (7849) and offers a link to this resource. Clicking on this link, the user learns that the official name of this gene is “paired box 8,” which is syntactically different from the short form “*PAX8*” found in the text. However, when a user searches with the NCBI Gene ID 7849 in *Pubtator*, all abstracts mentioning any of the synonyms of this gene will be returned. This functionality reduces the risk of missing important synonyms and relieves a user from formulating long queries composed of terms that are connected by the Boolean operator OR.

**Figure 4:**
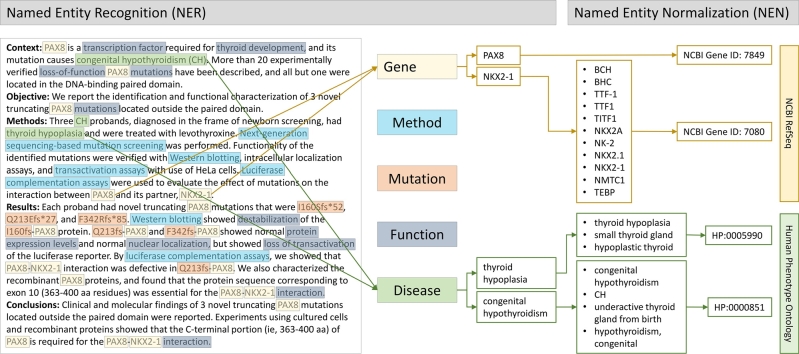
**Downstream processing of text mentions**. Further processing of the abstract from Figure 3B for named entity recognition (NER) and named entity normalization (NEN) for the mentions of “genes” and “diseases” as examples. Both tasks are achieved by using lists of synonyms and of ontologies such as the *NCBI Refseq* [18] and the *Human Phenotype Ontology* (HPO) [19].

The user must be aware that this recognition and mapping is computed automatically and is not error-free. The quality of recognition and mapping across all *PubMed* abstracts is difficult to assess and is different for different types of entities. While the quality for human genes or human diseases can be expected to be rather high (80–90 %), detection and normalization of sequence variants or chemicals is considerably more difficult [20]. Especially articles that contain gene symbols from different species, in particular from humans and from other mammals that often share identical gene symbols, often exhibit wrong mappings because they cannot be disambiguated correctly with respect to the species in which a specific finding was made.

## Tools for information extraction

While search engines identify articles relevant for a given information request, the user has to capture the concrete knowledge within each article by reading. For large search results, this becomes a tedious and time-consuming effort. Automatic IE, i. e., the application of software tools to extract specific information from large collections of articles, is a subject of intense research [21]. Though current IE tools are still in the prototype stage and their use requires advanced computational skills, IE applications are beginning to be included into biomedical knowledge management pipelines and tools. For instance, *Pubtator* uses IE tools to identify and highlight important entities. For the construction of curated databases, IE is used to economize the screening of large document collections.

IE consists of three main steps: **(i)** determination, download, and pre-processing of all articles that are to be analyzed, e. g., all articles mentioning a certain disease. **(ii)** Recognition of specific entities considered as relevant for the task, e. g., all mentions of TFBSs and of genetic variants. This process is called *named entity recognition* (NER) [22]. **(iii)** *Named entity normalization* (NEN) [23] then associates the recognized entities to a unique identifier from a reference database, e. g., gene and gene product mentions are linked to their entry in the NCBI Gene database (see Table 2C, Figure 4). While NER identifies occurrences of relevant information, NEN makes this information comparable over different articles each using its own nomenclature. For instance, the gene *BRCA1*, corresponding to the NCBI Gene ID 672, is also known under the synonyms *IRIS*, *PSCP*, or *FANCS*. NEN ensures that mentions of any of these names are recognized as referring to the same gene (Figure 4).

State-of-the-art systems for all three steps use advanced statistical natural language processing and deep learning algorithms [17]. Two examples of high-quality frameworks with simple programming interfaces for NER are *HunFlair* [24] and *SciSpacy* [25]. *HunFlair* is a recent framework achieving the to-date best accuracy for the detection of genes, proteins, species, diseases, chemicals, and cell lines. *SciSpacy* offers a broader functionality, but achieves a lower quality for IE in biomedicine. Both frameworks are available libraries for the Python programming language, but neither contains algorithms for the considerably harder NEN step. For this, developers must resort to other tools, such as *BERN* [26], *tmChem* [27], or *TaggerOne* [28].

More and more IE tools emerge that specialize in the detection and normalization of regulatory features. Examples are *HistoNER*, a tool for the extraction of histone modifications [29], and *tmVAR* [30], which detects mentions of protein and gene variants. We recently developed a method for the extraction of regulatory relationships between TFs and genes in human cells [31].

## Literature-based curated data sources

Once an algorithm for the extraction of specific information from scientific articles has been developed, it can be applied to large text collections, provided sufficient computational resources are available. A typical pipeline for large-scale application of IE comprises the following steps: **(i)** application of the IE system to all articles in *PubMed*, **(ii)** discarding all extracted information already present in known databases, and **(iii)** ranking all new information by the system’s confidence prediction and determination of a threshold to remove lower-quality data. The remaining data items can either be used directly for downstream analysis or undergo expert curation to further increase their reliability. For instance, the enhancer database *EnDB* [32] was created by **(i)** identifying all *Medline* abstracts mentioning an enhancer, **(ii)** annotating these entities with genes, binding sites, and DNA variants, and **(iii)** subsequent human verification of the extracted information and their logical associations.

Table 2C depicts a list of databases that have been created by similar pipelines. Their content is either manually curated or was derived by semi-automatic methods. Users can expect a substantially wider coverage of resources extracted by semi-automatic methods, yet must pay more attention to the correctness of the extracted data.

The development of new IE tools and pipelines for their large-scale application to biomedical texts requires computational skills in the areas of machine learning, big data processing, and natural language processing. In the field of regulatory features of the non-coding genome, the DFG Research Unit FOR2841 “*Beyond the Exome*” aims to develop such systems for the detection of enhancers, TFs, histone modifications, and genetic variants and for the investigation of their associations with genetic diseases. Results will be made freely available to the scientific community through a future version of our *RegulationSpotter*^25^ software.

## Conclusions

Retrieving data about functional DNA features from large high-throughput projects such as *ENCODE* is easy *via* user-friendly databases and web interfaces. Some of the data are integrated in metadatabases such as *Ensembl* or the *UCSC*. Accessing data from individual disease-driven studies is much more difficult. Dedicated search engines can help to find relevant articles. IE tools allow for automatic processing of the results, though these tools are currently difficult to apply to specific cases. As the study of the non-coding genome and IE are in rapid progress, we foresee that automatic analysis of the scientific literature will become more relevant for the integration of new knowledge into existing resources.

## Footnotes

1
https://www.omim.org/

2
https://www.encodeproject.org/

3
https://www.blueprint-epigenome.eu/

4
https://fantom.gsc.riken.jp/5/

5
https://gtexportal.org/home/

6
http://www.roadmapepigenomics.org/

7
https://www.ensembl.org/index.html

8
https://genome.ucsc.edu/

9
https://m.ensembl.org/info/data/biomart/index.html

10
https://www.encodeproject.org/

11
http://www.roadmapepigenomics.org

12
https://fantom.gsc.riken.jp

13
https://grch37.ensembl.org/info/genome/funcgen/index.html

14
https://www.ucsc.edu/

15
http://www.licpathway.net/ENdb/index.php

16
http://bioinfo.au.tsinghua.edu.cn/endisease/

17
http://biocc.hrbmu.edu.cn/DiseaseEnhancer

18
http://jaspar.genereg.net/

19
http://gtrd.biouml.org/#!

20
http://www.oreganno.org

21
https://www.regulomedb.org/regulome-search/

22
https://www.nlm.nih.gov/bsd/medline.html

23
https://www.ncbi.nlm.nih.gov/research/pubtator

24
https://www.ncbi.nlm.nih.gov/gene

25
https://www.regulationspotter.org/
